# A CRISPR-Based Toolbox for Studying T Cell Signal Transduction

**DOI:** 10.1155/2016/5052369

**Published:** 2016-02-01

**Authors:** Shen Chi, Arthur Weiss, Haopeng Wang

**Affiliations:** ^1^School of Life Science and Technology, ShanghaiTech University, Shanghai 200031, China; ^2^Division of Rheumatology, Department of Medicine, Rosalind Russell Medical Research Center for Arthritis, University of California, San Francisco, CA 94143, USA; ^3^Howard Hughes Medical Institute, University of California, San Francisco, CA 94143, USA

## Abstract

CRISPR/Cas9 system is a powerful technology to perform genome editing in a variety of cell types. To facilitate the application of Cas9 in mapping T cell signaling pathways, we generated a toolbox for large-scale genetic screens in human Jurkat T cells. The toolbox has three different Jurkat cell lines expressing distinct Cas9 variants, including wild-type Cas9, dCas9-KRAB, and sunCas9. We demonstrated that the toolbox allows us to rapidly disrupt endogenous gene expression at the DNA level and to efficiently repress or activate gene expression at the transcriptional level. The toolbox, in combination with multiple currently existing genome-wide sgRNA libraries, will be useful to systematically investigate T cell signal transduction using both loss-of-function and gain-of-function genetic screens.

## 1. Introduction

T cells play an essential role in the human immune system and have been identified as mediators of various systemic autoimmune diseases. In the past two decades, the studies of how T cell receptors (TCRs) are stimulated by non-self-antigens and how T cell activation is regulated are central topics in the immunology field [1, 2]. T cell stimulation is triggered by the engagement of the TCR to a cognate peptide-major histocompatibility complex (MHC) on antigen presenting cells (APCs). Following the formation of a TCR-peptide-MHC complex, two tyrosine residues, which are part of the immunoreceptor tyrosine-based activation motifs (ITAMs) within the short proximal cytoplasmic tails of their TCR-associated CD3 and *ζ*-chain subunits, are phosphorylated [1]. Such phosphorylation of ITAMs is mediated by the T cell Src-related protein tyrosine kinases (Src-PTKs) such as Lck. Phosphorylated ITAMs further recruit the cytoplasmic kinase ZAP-70 via its tandem Src-homology domains binding to the doubly phosphorylated ITAMs. ZAP-70 subsequently phosphorylates two signaling adaptor proteins, the linker for the activation of T cells (LAT) and the SH2-domain-containing leukocyte protein of 76 kDa (SLP-76). These two molecules function as the scaffolds to recruit many other signaling molecules, which eventually lead to T cell activation [2].

Most of our fundamental knowledge of TCR signaling transduction came from the studies of several human T cell lines, particularly the Jurkat leukaemic T cell line [3]. To investigate how signaling effector proteins mediate T cell activation response, a series of Jurkat mutants were generated by using mutagenesis. For example, J.CaM1 cell line is a Jurkat-derived Lck deficient mutant, which is impaired in T cell signaling [4]. This cell line was generated by sorting mutagenized Jurkat cells, which had a defective Ca^2+^ mobilization response triggered by TCR stimulation. Using a similar Ca^2+^ flux based strategy, the P116 cell line, which lacks ZAP-70 expression, was generated and has been widely used in studies of ZAP-70 function [5].

With the development of sequence-specific DNA nuclease technologies, genome editing is becoming a critical approach used to analyze biological functions in cell lines and animal models [6]. More recently, clustered regularly interspaced palindromic repeats- (CRISPR-) associated (Cas) protein 9 (known as CRISPR-Cas9) system has been demonstrated to target and induce site-specific DNA double-strand breaks (DSBs) directed by a single-guide RNA (sgRNA) [7]. The DNA repair of DSBs is mediated by either nonhomologous end joining (NHEJ) or homology directed repair (HDR), which can be used to introduce random or specific mutations, repair of endogenous mutations, or insertion of DNA elements [8]. Interestingly, the deactivated Cas9 (dCas9), which results from inactivated nuclease domains in Cas9, is also able to function as a DNA-binding scaffold to either silence (CRISPRi) or activate (CRISPRa) gene expression, depending on the transcription effector domains fused to Cas9 [9–11]. Consequently, the CRISPR-Cas9 system has been widely used to precisely and effectively generate engineered eukaryotic cells [12–14]. Recently, a series of studies performed both loss-of-function and gain-of-function screens in a genome-wide scale in* E. coli* [15], zebrafish cells [16], and K562 tumor cell lines [17] as well as primary mouse dendritic cells [18]. In addition, several human sgRNA libraries for genome-wide screen have been established [10, 19, 20]. However, to our knowledge, a CRISPR-based genome-wide screen to study T cell activation has not been reported, which might be largely due to a lack of Jurkat cell lines optimized for such screens. Here we developed a toolbox of three Jurkat cell lines, which are engineered for CRISPR, CRISPRi, or CRISPRa screens, respectively. These cell lines were derived from a single cell clone and expressed uniform and normal levels of TCR and CD28 receptors to ensure they could undergo efficient T cell stimulation. We also demonstrated that we could use CRISPR, CRISPRi, and CRISPRa to target endogenous genes and regulate their expression levels in these cell lines. Collectively, this toolbox represents a useful platform for systematically dissecting T cell signaling pathways.

## 2. Results

**Figure 1:**
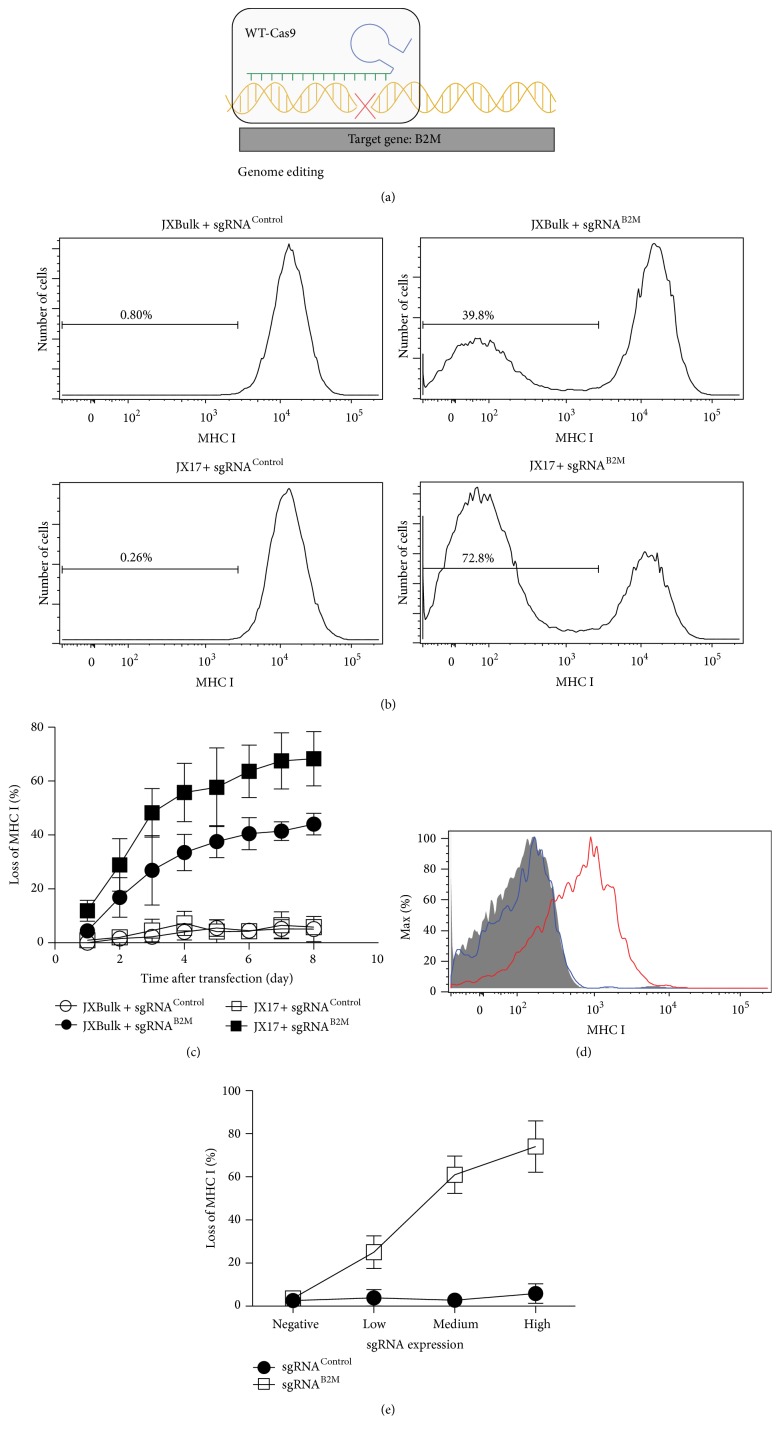
A Jurkat T cell line optimized for WT-Cas9 mediated genome editing. (a) WT-Cas9 generates DNA double-strand breaks at the targeted genome locus, resulting in disruption of the target gene. (b) JX17 cells achieve high genome editing efficiency. Jurkat cells stably expressing WT-Cas9 protein were transfected with constructs expressing the sgRNA^Control^ or the sgRNA^B2M^. Cells were grown for 6 days and then analyzed for MHC I expression in the GFP+ transfected cells. Data are shown in histogram and are representative of four independent experiments. (c) Disruption of gene by WT-Cas9 is irreversible. Jurkat cells were transfected with sgRNAs as described in (b). The expression of MHC class I was assessed by FACS at different time points after transfection. The chart summarizes the results of three independent experiments (data represent the mean value ± SD). (d) Loss of MHC class I expression was restored by exogenous expression of B2M gene. JX17 cells were electroporated with sgRNA^B2M^ as described in (b). MHC class I-negative JX17 cells were sorted and electroporated with either an empty vector (blue histogram) or a plasmid expressing B2M gene (red histogram). The expression of MHC class I was assessed by FACS 48 hours after electroporation. The grey histogram represents the negative control (unstained sample). (e) WT-Cas9 edits genome in an sgRNA dose-dependent manner. The transfected cells were divided into four populations according to their GFP expression. The percentage of cells losing MHC I expression was quantified by flow cytometry 6 days following transfection. The chart summarizes the results of three independent experiments (data represent mean value ± SD).

The CRISPR-Cas9 system has proven to be a powerful tool to perform individual gene editing and large-scale genetic screens [19] (Figure 1(a)). Recently, the CRISPR/Cas9 system has been used in Jurkat T cells as well as primary human T cells [21–24]. However, to our knowledge, no Cas9-based loss-of-function genetic screen has been reported in human T cells, probably due to the difficulty of expressing functional Cas9 within T cells. To facilitate future genetic screen using human T cells, we sought to generate a Jurkat cell line stably expressing functional WT-Cas9 and optimized for large-scale genetic screens.

We first cloned wild-type Cas9 and a 2A-linked blue florescent protein (BFP) reporter gene into a lentiviral construct driven by the spleen focus-forming virus promoter (SFFV). This lentivirus was generated and used to infect Jurkat T cells. After lentiviral transduction, flow cytometry sorting was used to isolate a bulk population of BFP-positive Jurkat cells with normal surface expression amounts of both TCR and CD28 receptors, two major cell surface receptors that contribute to T cell activation. We named this cell population as JXBulk. To test whether WT-Cas9 protein in JXBulk cells had any genome editing function, we designed an sgRNA to specifically target the beta-2 microglobulin (B2M) gene. B2M is a subunit of MHC class I molecules, which are highly expressed in all leukocytes, including human T cells. It has been demonstrated that disruption of B2M gene resulted in ablating MHC class I surface expression [25]. Therefore, the loss of MHC class I expression after expressing sgRNA against B2M served as a functional readout of WT-Cas9 activity in JXBulk cells. We expressed a control sgRNA (sgRNA^Control^) or an sgRNA targeting B2M gene (sgRNA^B2M^) in JXBulk cells by electroporation. The sgRNA expressing vector also contains the GFP gene as a reporter. We measured the expression level of surface MHC class I molecules in GFP+ JXBulk cells using FACS analysis. We found that there was little effect on MHC class I expression in the sample expressing sgRNA^Control^. In contrast, about 40% of JXBulk cells completely lost MHC class I expression 6 days after electroporation of sgRNA^B2M^ (Figure 1(b)). Our kinetic results also indicated that the disruption of MHC class I expression caused by WT-Cas9 mediated gene editing was permanent and irreversible (Figure 1(c)).

To further optimize the efficiency of WT-Cas9, JXBulk cells were single cell sorted and over 48 single cell subclones were further cultured. We compared the efficiency of WT-Cas9 function in these subclones. We found that clone 17 (hereafter referred to as JX17) displayed much higher gene editing efficiency than JXBulk. Our results show that more than 60% of JX17 cells lose their MHC class I expression 6 days after sgRNA^B2M^ electroporation (Figure 1(c)). Therefore, single cell sorting and subcloning are an effective approach to identify cells with optimal Cas9 efficiency. Our results also suggested that it required at least 6 days of cell growth after expression of sgRNA into JX17 cells to permit WT-Cas9 to reach maximal gene editing efficiency in a Cas9-based genetic screen and this should be considered for future genetic screens.

It is reported that wild-type Cas9 is able to cleave off-target sites that have up to five mismatches relative to the guide RNA [26, 27]. To test whether the loss of MHC class I expression we observed in Figure 1(b) was caused by off-target effects of wild-type Cas9 expressed in JX17, we performed a rescue experiment. Briefly, JX17 cells were electroporated with sgRNA^B2M^ as described in Figure 1(b), and we sorted MHC class I-negative JX17 cells, whose endogenous B2M gene was disrupted by electroporation of sgRNA^B2M^. We then expressed either an empty vector or the human B2M cDNA in these sorted cells by electroporation. 48 hours later, we measured surface expression of MHC class I by flow cytometry (Figure 1(d)). We found that exogenous expression of B2M gene, but not empty vector, increased MHC class I surface expression level, suggesting the loss of MHC class I expression indeed resulted from B2M deficiency.

We next tested whether the effectiveness of WT-Cas9 disruption of the target gene was related to the sgRNA dose. Six days after sgRNA transfection, the transfected JX17 cells were divided into four groups with negative, low, medium, or high expression of GFP, whose expression should be proportional to sgRNA level in cells. The expression of control sgRNA did not affect the surface level of MHC class I. In contrast, there was an sgRNA dose-dependent increase in the percentage of T cells that lost expression of surface MHC class I molecules. We found that an intermediate expression level of sgRNA was sufficient to reach maximal gene editing in JX17 cells (Figure 1(e)). Collectively, these results revealed the potential of JX19 cell line as a useful platform to perform WT-Cas9 mediated genetic screens.

**Figure 2:**
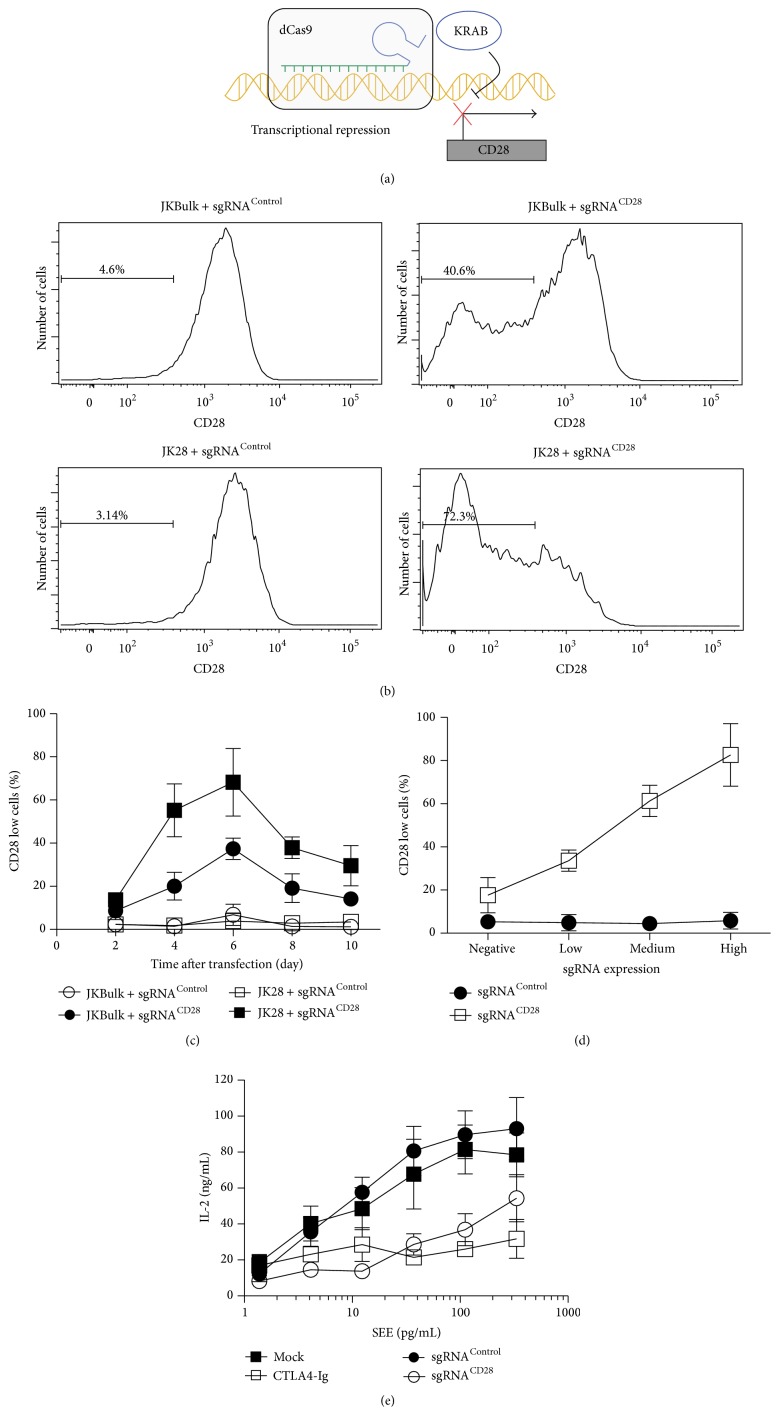
Engineering of A Jurkat T cell line for dCas9-KRAB mediated gene silencing. (a) In the CRISPRi system, dCas9 fused to KRAB domain can repress transcription of target gene. (b) sgRNA expression in JK28 cells can remarkably downregulate its targeting gene expression. Jurkat cells stably expressing dCas9-KRAB protein were transfected with constructs expressing sgRNA^Control^ or sgRNA^CD28^. Cells were grown for 6 days and then analyzed for CD28 expression in the GFP+ transfected cells. Data are shown in histogram and are representative of three independent experiments. (c) Transcription repression mediated by dCas9-KRAB is reversible. JKBulk and JK28 cells were transfected with sgRNAs as described in (b). The percentages of the cells with reduced expression of CD28 were assessed by FACS at different time points after transfection. The data summarize the results of three independent experiments (data represent mean value ± SD). (d) sgRNA expression is a limiting factor for CRISPRi function. The transfected cells were divided into four populations according to their GFP expression. The percentage of cells losing CD28 expression was quantified by flow cytometry 6 days following transfection. The data summarizes the results of three independent experiments (data represent mean value ± SD). (e) Silencing CD28 expression by CRISPRi interference IL-2 production in activated T cells. IL-2 assays were performed in the indicated conditions. The data summarize the results of three independent experiments (data represent mean ± SD).

CRISPR interference (CRISPRi) has been demonstrated to be a robust system to turn down gene expression at the transcriptional level with minimal off-target effects [28]. The Krüppel associated box (KRAB) domain, a transcriptional repression domain, is often fused to dCAS9 and used in CRISPRi systems (Figure 2(a)) [29]. In addition to repressing coding RNAs, CRISPRi can also target transcripts including noncoding RNAs and microRNAs [30]. However, a T cell line specifically optimized for the CRISPRi system has not been established. To address this issue, we stably expressed a dCas9-BFP-KRAB fusion protein, which was described previously [10], in Jurkat T cells. We sorted BFP+Jurkat cells expressing dCas9-KRAB (hereafter referred to as JKBulk) with high expression of TCR and CD28 receptors. To test the activity of dCas9-KRAB in JKBulk, we transfected an sgRNA targeting the CD28 gene and measured surface CD28 expression at different time points following transfection (Figures 2(b) and 2(c)). Similar to the WT-Cas9 system, we also used single cell sorting to identify a subclone cell line (JK28) with optimal dCas9-KRAB activity. We observed that about 40% of JKBulk cells and approximately 70% of JK28 cells had reduced CD28 expression amounts 6 days after transfection (Figure 2(b)). Interestingly, unlike the WT-Cas9-based genomic DNA editing, the reduction of CD28 expression was gradually recovered, which might result from the degradation of the CD28 sgRNA as the cells were cultured following transient transfection of the CD28 sgRNA (Figure 2(c)). Our results suggested that stable expression of sgRNA might be needed to maintain dCas9-KRAB mediated gene silencing. Hence, delivery of sgRNA via viral transduction might be a means to achieve long time gene silencing in JK28 cells.

To test whether the sgRNA is a limiting factor for CRISPRi activity, we divided the transfected JK28 cells into four populations with GFP negative, low, medium, or high expression level. We measured surface expression level of CD28 by flow cytometry 6 days following transfection (Figure 2(c)).  Figure 2(d) revealed a dose relationship between the sgRNA expression and the percentage of cells with low expression of CD28. Notably, over 80% of cells lose CD28 expression in the population with high GFP expression level. Thus, robust expression of sgRNA is likely preferable in a CRISPRi based genetic screen.

We next aimed to test whether gene silencing using dCas9-KRAB could induce a functional change of the JK28 cell line. CD28 is a costimulatory receptor that can provide a second signal, when combined with TCR ligands, to induce interleukin 2 (IL-2) production [31]. Blocking CD28 function by using CTLA4-Ig, a CTLA4-immunoglobulin recombinant fusion protein, during Jurkat T cell activation largely inhibited the production of the cytokine IL-2 (Figure 2(e)) [31]. We tested whether transfected sgRNA targeting CD28 in JK28 cells could result in a similar effect. We found that silencing CD28 expression using dCas9-KRAB markedly reduced IL-2 production. In contrast, JK28 cells expressing control sgRNA did not affect the amount of IL-2 production. This result demonstrated that dCas9-KRAB in JK28 cells potently mediated gene silencing that could result in alteration of Jurkat T cell function.

**Figure 3:**
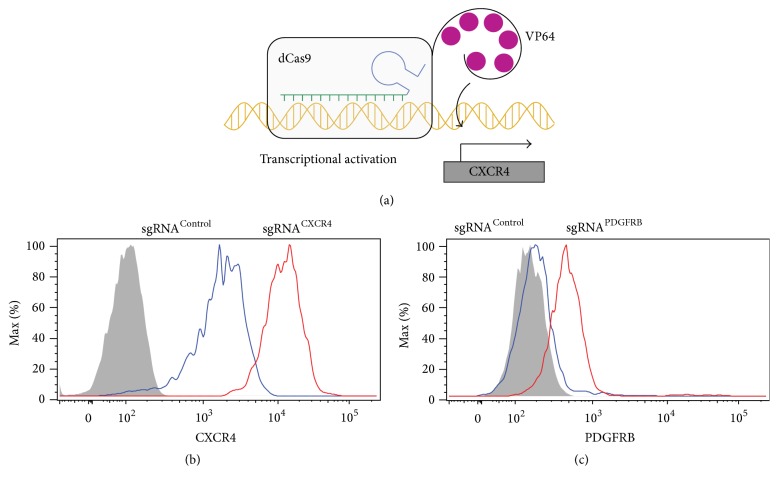
A Jurkat T cell line with sunCas9 system. (a) In the CRISPRa system, dCas9 fused to SunTag is able to recruit multiple copies of VP64 and can activate gene transcription. (b-c) sgRNA expressions in JS19 cells can substantially upregulate their targeting gene expressions. JS19 cells were transduced with virus expressing the sgRNA^Control^ (blue histograms), the sgRNA^CXCR4^ (red histogram in (b)), or the sgRNA^PDGFRB^ (red histograms in (c)). Cells were grown for more than a week and then analyzed for CXCR4 expression (b) and PDGFRB expression (c) in the transduced cells. The grey histograms represent the negative control (unstained sample). Data are shown in histograms and are representative of three independent experiments.

Recently, Tanenbaum et al. developed a sunCas9 system, which could be used to activate gene transcription (CRISPRa) [11]. In this system, multiple copies of the transcriptional activating effector domain (VP64) are recruited to a single dCas9 protein fused with the SunTag. The sgRNA targets the sunCas9 system to the promoter region of an endogenous gene and turns on its transcription (Figure 3(a)). To generate a T cell line with functional sunCas9, Jurkat cell lines were generated to express both dCas9-SunTag-P2A-BFP and GCN4-sfGFP-NLS-VP64, which were described previously [11]. Transduced GFP+BFP+Jurkat cells with high-level expression of TCR and CD28 receptors were single cell sorted and 24 single cell subclones were further cultured. To test the sunCas9 function in transduced cells, these cells were infected with a lentivirus that expressed either an sgRNA^control^ or an sgRNA targeting CXCR4 (sgRNA^CXCR4^). CXCR4 is a chemokine receptor that is highly expressed in T cells and plays important roles in T cell trafficking and homing. We measured the CXCR4 expression using flow cytometry 6 days after lentivirus infection. Among 24 single cell subclones we screened, one clone (JS19) has the highest activity of sunCas9 system. In JS19 cells, we observed a remarkable upregulation of CXCR4 expression (6-fold) in JS19 cells expressing sgRNA^CXCR4^ compared to those infected with sgRNA^Control^ virus (Figure 3(b)). CXCR4 is well-expressed in Jurkat cells (Figure 3(b)), so we next tested whether a gene that is normally not expressed in T cells could be induced using JS19 cell line. To this end, we chose the platelet-derived growth factor receptor (PDGFR), which is a cell surface tyrosine kinase growth factor receptor for members of the platelet-derived growth factor family [32]. Jurkat cells normally are negative for PDGFR expression [33]. Consistent with this, we detected little expression of PDGFRB in JS19 cells expressing sgRNA^Control^. In contrast, transduction of sgRNA targeting PDGFRB substantially enhanced PDGFR expression level in JS19 cells. Taken together, these results showed that sunCas9 system in JS19 robustly increased the transcription of an endogenous gene and could be a useful platform to perform a gain-of-function genetic screen in human T cell lines.

## 3. Discussion

Systematic investigation of a signaling pathway depends on the ability to manipulate potential effector genes involved within this pathway through deletion, suppression, or overexpression. To study T cell signaling transduction, we developed a toolbox including three human T cell lines, JX17, JK28, and JS19. Using this toolkit, it should be possible to perform both loss-of-function and gain-of-function genetic screens in the Jurkat-derived human T cell lines. To help researchers determine which cell line they should use in their future screens, we compared these three cell lines based on our results as well as previous studies [6, 10] and summarized results in Table 1.

Notably, our toolkit has the following unique advantages. First, these three cell lines were sorted to ensure they expressed high level of the TCR and CD28. Therefore, these cell lines could undergo efficient T cell activation; for example, JK28 was able to produce a substantial amount of IL-2 after T cell activation (Figure 2(e)). Second, all three cell lines are derived from the same population of Jurkat cells maintained in our lab. Though expressing different Cas9 variants, there is minimal internal variation among these cell lines. Thus, the results generated from different genetic screens can be compared in a similar context. Lastly, we should be able to combine multiple genetic screens using this toolbox to address the same biological question at the same time. For example, two independent loss-of-function screens, one using JX17 cells expressing WT-Cas9 and the other using JK28 cells expressing dCas9-KRAB, could be performed side by side. We would expect to identify the candidate genes with high confidence. On the other hand, researchers will be able to use JK28 cells (CRISPRi) and JS19 cells (CRISPRa) to screen for both loss-of-function and gain-of-function phenotypes in the same biological assay. These two screen results should complement each other to provide a rich and comprehensive understanding of T cell signaling.

In sum, we believe that our toolbox will be a useful platform to study T cell function. The programmability of Cas9 and extensive protein engineering of Cas9 will lead to the development of more Cas9 variants with novel functions. We expect to further increase family members in our toolbox in the future.

## 4. Experimental Procedures

### 4.1. Cell Culture and Reagent

Jurkat cells were grown in RPMI-1640 containing 5% FCS/Pen/Strep. Anti-HLA-PE-Cy7 (clone: G46-2.6), anti-CXCR4-APC (clone: 12G5), and anti-PDGFRB (clone: REA363) were purchased from BD, Biolegend and Miltenyi Biotech, respectively.

### 4.2. sgRNA Sequence Selection

For sgRNA^B2M^, we manually chose a 20 nt guide sequence preceding the 5′-NGG PAM, which contains the ATG start codon of B2M gene. For the sgRNA used in CRISPRi system, we followed the rule that the sgRNA should be located within a region from −50 to +300 bp relative to the transcriptional starting site (TSS) [34]. Since the TSS site of the human CD28 gene was previously mapped to between +1 and +61 of the 1st exon [35] and ATG start codon located at +223, we chose an sgRNA sequence around the ATG start codon for the convenience. For sgRNA^CXCR4^, we used an sgRNA sequence that was validated in the previous study [11]. The sgRNA^PDGFRB^ and sgRNA^Control^ were generously provided by Dr. Boettcher at UCSF from an sgRNA library (unpublished data). The following sgRNA were used:

 sgRNA^Control^: GTAGAACGAGCCAACCATTT,
 sgRNA^B2M^: GGCCGAGATGTCTCGCTCCG,
 sgRNA^CD28^: GAGCATCTTTGTCCTGACGA,
 sgRNA^CXCR4^: GCAGACGCGAGGAAGGAGGGCGC,
 sgRNA^PDGFRB^: ATCCTGAGCGAACGGGCGAT.

### 4.3. Generation of Jurkat Cells Stably Expressing Cas9 Variants

We fused the WT-Cas9 that was flanked by two nuclear localization sequences (NLS) with T2A-BFP and inserted this cassette into a lentivirus vector under SFFV promoter. The dCas9-KRAB and dCas9-SunTag as well as scFV-sfGFP-VP64 constructs were described previously [10, 11]. Lentivirus expressing Cas9 variants was generated using 293FT cells. The WT Jurkat T cells were expanded in the medium containing the virus for a week and these cells were harvested and stained with anti-CD28 (Tonbo, clone: CD28.2) and anti-CD3 (Tonbo, clone: UCHT1). The BFP+CD28+CD3+ Jurkat cells were then isolated using flow cytometry for further culture. For the sunCas9 system, Jurkat cells were transduced with both dCas9-SunTag virus and scFV-sfGFP-VP64 virus. One week after transduction, BFP+GFP+CD28+CD3+Jurkat cells were sorted. Upon request, all of the three cell lines (JX17, JK28, and JK19) are available from Dr. Haopeng Wang's lab at ShanghaiTech University (wanghp@shanghaitech.edu.cn).

### 4.4. Expression of sgRNA in Jurkat Cells by Electroporation or Lentiviral Transduction

The sgRNAs were cloned into pLKO.1-GFP vector (a gift from Dr. Shen from NIBS) [36]. To facilitate FACS-based stable clone selection, the original pLKO.1-puro plasmid (Addgene# 8453) was modified. Briefly, EGFP was cloned into the original pLKO vector to replace the puroR sequence using BamHI and KpnI, and then DNA sequence containing gRNA scaffold was cloned into a site following the U6 promoter using NdeI and EcoRI. sgRNA sequence was inserted into the pLKO.1-GFP vector by using the BfuAI site. This vector is available upon request by sending an email to Dr. Xiaodong Wang (wangxiaodong@nibs.ac.cn) or Dr. Zhirong Shen (shenzhirong@nibs.ac.cn). Jurkat cells were transfected with these sgRNA plasmids using electroporation according to the preset protocol in Genepulser (Bio-Rad). At different time points after transfection, Jurkat cells were harvested and analyzed by using BD Fortessa flow cytometer. For the sunCas9 system, JS19 cells were infected with lentivirus encoding the sgRNA. Transduced cells were cultured for a week. Measurements of surface CXCR4 level were performed by FACS.

### 4.5. IL-2 Assay

Jurkat T cells were stimulated by superantigen SEE presented by Raji B cells in the presence or the absence of CTLA4-Ig as described [31]. After 22 hours, the supernatants were collected and IL-2 production was measured by using Human IL-2 ELISA Kit (BD Biosciences) according to the manufacturer's instructions.

## Figures and Tables

**Table 1:** Comparisons between JX17, JK28, and JS19.

	JX17	JK28	JS19
Genetic screen type	Loss-of-function	Loss-of-function	Gain-of-function
Mechanism	Frame shift genome mutation	Transcriptional repression	Transcriptional activation
Regulation	Permanent	Reversible	Reversible^*∗*^
Target region	Gene^*∗*^	Gene, microRNA, and lincRNA^*∗*^	Gene, microRNA, and lincRNA^*∗*^
Efficiency	>70%	>80%	3–6-fold
sgRNA expression time required	>5 days	>6 days	A week
Available genome-wide libraries	Ref. [19, 20]	Ref. [10]	Ref. [10]

^*∗*^Based on [6].

## References

[1] Chakraborty A. K., Weiss A. (2014). Insights into the initiation of TCR signaling. *Nature Immunology*.

[2] Wang H., Kadlecek T. A., Au-Yeung B. B. (2010). ZAP-70: an essential kinase in T-cell signaling. *Cold Spring Harbor perspectives in biology*.

[3] Abraham R. T., Weiss A. (2004). Jurkat T cells and development of the T-cell receptor signalling paradigm. *Nature Reviews Immunology*.

[4] Goldsmith M. A., Weiss A. (1987). Isolation and characterization of a T-lymphocyte somatic mutant with altered signal transduction by the antigen receptor. *Proceedings of the National Academy of Sciences of the United States of America*.

[5] Williams B. L., Schreiber K. L., Zhang W. (1998). Genetic evidence for differential coupling of Syk family kinases to the T-cell receptor: reconstitution studies in a ZAP-70-deficient Jurkat T-cell line. *Molecular and Cellular Biology*.

[6] Boettcher M., McManus M. T. (2015). Choosing the right tool for the job: RNAi, TALEN, or CRISPR. *Molecular Cell*.

[7] Jinek M., Chylinski K., Fonfara I., Hauer M., Doudna J. A., Charpentier E. (2012). A programmable dual-RNA-guided DNA endonuclease in adaptive bacterial immunity. *Science*.

[8] Doudna J. A., Charpentier E. (2014). The new frontier of genome engineering with CRISPR-Cas9. *Science*.

[9] Gilbert L. A., Larson M. H., Morsut L. (2013). CRISPR-mediated modular RNA-guided regulation of transcription in eukaryotes. *Cell*.

[10] Gilbert L. A., Horlbeck M. A., Adamson B. (2014). Genome-scale CRISPR-mediated control of gene repression and activation. *Cell*.

[11] Tanenbaum M. E., Gilbert L. A., Qi L. S., Weissman J. S., Vale R. D. (2014). A protein-tagging system for signal amplification in gene expression and fluorescence imaging. *Cell*.

[12] Ran F. A., Hsu P. D., Wright J., Agarwala V., Scott D. A., Zhang F. (2013). Genome engineering using the CRISPR-Cas9 system. *Nature Protocols*.

[13] Hsu P. D., Lander E. S., Zhang F. (2014). Development and applications of CRISPR-Cas9 for genome engineering. *Cell*.

[14] Zalatan J. G., Lee M. E., Almeida R. (2015). Engineering complex synthetic transcriptional programs with CRISPR RNA scaffolds. *Cell*.

[15] Bikard D., Jiang W., Samai P., Hochschild A., Zhang F., Marraffini L. A. (2013). Programmable repression and activation of bacterial gene expression using an engineered CRISPR-Cas system. *Nucleic Acids Research*.

[16] Jao L.-E., Wente S. R., Chen W. (2013). Efficient multiplex biallelic zebrafish genome editing using a CRISPR nuclease system. *Proceedings of the National Academy of Sciences of the United States of America*.

[17] Mali P., Yang L., Esvelt K. M. (2013). RNA-guided human genome engineering via Cas9. *Science*.

[18] Parnas O., Jovanovic M., Eisenhaure T. M. (2015). A genome-wide CRISPR screen in primary immune cells to dissect regulatory networks. *Cell*.

[19] Shalem O., Sanjana N. E., Hartenian E. (2014). Genome-scale CRISPR-Cas9 knockout screening in human cells. *Science*.

[20] Wang T., Wei J. J., Sabatini D. M., Lander E. S. (2014). Genetic screens in human cells using the CRISPR-Cas9 system. *Science*.

[21] Liang X., Potter J., Kumar S. (2015). Rapid and highly efficient mammalian cell engineering via Cas9 protein transfection. *Journal of Biotechnology*.

[22] Hendel A., Bak R. O., Clark J. T. (2015). Chemically modified guide RNAs enhance CRISPR-Cas genome editing in human primary cells. *Nature Biotechnology*.

[23] Schumann K., Lin S., Boyer E. (2015). Generation of knock-in primary human T cells using Cas9 ribonucleoproteins. *Proceedings of the National Academy of Sciences*.

[24] Li C., Griffin G. E., Liu Y. (2015). Inhibition of HIV-1 infection of primary CD4^+^ T-cells by gene editing of CCR5 using adenovirus-delivered CRISPR/Cas9. *Journal of General Virology*.

[25] Riolobos L., Hirata R. K., Turtle C. J. (2013). HLA engineering of human pluripotent stem cells. *Molecular Therapy*.

[26] Fu Y., Foden J. A., Khayter C. (2013). High-frequency off-target mutagenesis induced by CRISPR-Cas nucleases in human cells. *Nature Biotechnology*.

[27] Kuscu C., Arslan S., Singh R., Thorpe J., Adli M. (2014). Genome-wide analysis reveals characteristics of off-target sites bound by the Cas9 endonuclease. *Nature Biotechnology*.

[28] Qi L. S., Larson M. H., Gilbert L. A. (2013). Repurposing CRISPR as an RNA-guided platform for sequence-specific control of gene expression. *Cell*.

[29] Larson M. H., Gilbert L. A., Wang X., Lim W. A., Weissman J. S., Qi L. S. (2013). CRISPR interference (CRISPRi) for sequence-specific control of gene expression. *Nature Protocols*.

[30] Kampmann M., Horlbeck M. A., Chen Y. (2015). Next-generation libraries for robust RNA interference-based genome-wide screens. *Proceedings of the National Academy of Sciences of the United States of America*.

[31] Tian R., Wang H., Gish G. D. (2015). Combinatorial proteomic analysis of intercellular signaling applied to the CD28 T-cell costimulatory receptor. *Proceedings of the National Academy of Sciences*.

[32] Aricò A., Guadagnin E., Ferraresso S. (2014). Platelet-derived growth factors and receptors in canine lymphoma. *Journal of Comparative Pathology*.

[33] Roose J. P., Diehn M., Tomlinson M. G. (2003). T cell receptor-independent basal signaling via Erk and Abl kinases suppresses RAG gene expression. *PLoS Biology*.

[34] Shalem O., Sanjana N. E., Zhang F. (2015). High-throughput functional genomics using CRISPR-Cas9. *Nature Reviews Genetics*.

[35] Lee K. P., Taylor C., Petryniak B., Turka L. A., June C. H., Thompson C. B. (1990). The genomic organization of the CD28 gene: implications for the regulation of CD28 mRNA expression and heterogeneity. *Journal of Immunology*.

[36] Jiang X., Jiang H., Shen Z., Wang X. (2014). Activation of mitochondrial protease OMA1 by Bax and Bak promotes cytochrome c release during apoptosis. *Proceedings of the National Academy of Sciences of the United States of America*.

